# Engineered Brimonidine Tartrate Aminosomes as a Mucoadhesive Platform for Sustained Glaucoma Management: Optimization, In Vitro Characterization, and In Vivo Evaluation

**DOI:** 10.3390/pharmaceutics18040422

**Published:** 2026-03-30

**Authors:** Waad M. Omar, Rodayna A. Shalaby, Osama Saher, Asmaa Ashraf Nemr, Ahmed M. Fatouh

**Affiliations:** 1 Department of Pharmaceutics and Industrial Pharmacy, School of Pharmacy, Newgiza University, Km. 22 Cairo-Alex Road, Giza P.O. Box 12577, Egypt; waad.mohamed@ngu.edu.eg (W.M.O.); ratef@ngu.edu.eg (R.A.S.); ahmed.abdelbar@ngu.edu.eg (A.M.F.); 2 Department of Pharmaceutics and Industrial Pharmacy, Faculty of Pharmacy, Cairo University, Kasr El-Ainy Street, Cairo P.O. Box 11562, Egypt; asmaa.nemr@pharma.cu.edu.eg; 3 Department of Laboratory Medicine, Karolinska Institute, P.O. Box 14152 Stockholm, Sweden; 4 Department of Cellular Therapy and Allogeneic Stem Cell Transplantation (CAST), Karolinska University Hospital Huddinge and Karolinska Comprehensive Cancer Center, P.O. Box 14152 Stockholm, Sweden

**Keywords:** Brimonidine Tartrate, glaucoma, mucoadhesive, Aminosomes, stearylamine, ocular drug delivery

## Abstract

**Purpose:** The objective of this study was to engineer and optimize a mucoadhesive, positively charged stearylamine-enriched liposomal platform, termed Aminosomes, to circumvent the biophysical barriers limiting the ocular bioavailability of Brimonidine Tartrate (BT), an alpha-2 adrenergic receptor agonist for glaucoma management. **Methods:** Aminosomes were synthesized using a tailored ethanol injection technique and optimized via a 3^2^ × 2^1^ full factorial design. Molecular integrity and crystallinity were assessed using Fourier-transform infrared spectroscopy (FTIR) and X-ray diffraction (XRD). The mucoadhesive potential was validated through a mucin interaction assay based on zeta potential shifts. In vitro release kinetics were evaluated using the dialysis membrane diffusion technique, while the therapeutic potential and ocular safety were validated through in vivo pharmacodynamic profiling of intraocular pressure (IOP) reduction, alongside comprehensive biocompatibility assessments via Draize irritancy protocol and histopathological examination. **Results:** The optimized Aminosomes exhibited nanometric dimensions, monodisperse size distribution, robust positive surface charge, and superior drug loading. FTIR and XRD analyses confirmed the chemical compatibility of the formulation components, as well as the successful encapsulation of BT and its transition to an amorphous state within the lipidic matrix. The mucoadhesion test demonstrated a high binding affinity for mucin. The in vitro release profile demonstrated a sustained-release pattern (78.8% over 12 h). Non-compartmental pharmacodynamic analysis of IOP-reduction data revealed a 2.8-fold increase in AUC_0–24h_, 3.5-fold extension in t_1/2_, and 5.2-fold prolongation in mean residence time (MRT) relative to the standard solution. **Conclusions:** The optimized Aminosomes demonstrated superior mucoadhesive anchoring, enhanced and sustained therapeutic flux without inducing ocular toxicity, offering a robust strategy for enhancing the pharmacodynamics of BT.

## 1. Introduction

The delivery of ophthalmic pharmacotherapeutics is fundamentally constrained by a sophisticated network of anatomical and physiological defense mechanisms designed to maintain ocular homeostasis by sequestering the internal environment from exogenous substances. These biological barriers significantly attenuate the bioavailability of topically applied medications, necessitating high-frequency dosing regimens that often lead to diminished patient compliance and potential systemic toxicity [1,2]. The ocular barrier system is bifurcated into static anatomical components and dynamic physiological processes. The anatomical barriers include the stratified corneal epithelium, conjunctiva, sclera, and the highly restrictive blood-aqueous and blood-retinal barriers, which collectively function as physical sieves and metabolic filters [3,4]. Conversely, the dynamic physiological processes involve rapid nasolacrimal drainage, reflexive blinking, and continuous tear film turnover, which synergistically promote the rapid clearance of topically instilled formulations [5,6]. Collectively, these mechanisms, supplemented by local lymphatic and hemic clearance, restrict the fraction of the administered dose capable of reaching targeted intraocular tissues [7]. Consequently, achieving therapeutic concentrations remains a paramount challenge in the management of chronic conditions such as glaucoma, a progressive optic neuropathy characterized by pathologically elevated intraocular pressure (IOP) and irreversible optic nerve head damage [8,9]. Effective clinical intervention requires the development of advanced delivery platforms capable of surmounting these formidable barriers to provide sustained modulation of aqueous humor dynamics [10,11].

Brimonidine Tartrate (BT), a highly selective alpha-2 adrenergic agonist, remains a cornerstone in the pharmacological management of ocular hypertension and glaucoma [12]. Its therapeutic efficacy is derived from a distinctive dual mechanism of action: the suppression of aqueous humor synthesis via the ciliary epithelium and the concomitant augmentation of uveoscleral outflow [13]. Despite its potency, the clinical utility of BT is severely compromised by its unfavorable physicochemical profile and rapid pharmacokinetic elimination. As a hydrophilic moiety, BT exhibits poor passive diffusion across the lipophilic corneal epithelium, which, when coupled with aggressive precorneal clearance, results in a suboptimal intraocular half-life of approximately 3 h [14]. Consequently, conventional therapy necessitates frequent daily administrations to maintain therapeutic thresholds, a regimen that frequently precipitates dose-dependent adverse effects, such as follicular conjunctivitis and conjunctival hyperemia, while significantly undermining patient adherence [15].

To address these limitations, recent research has pivoted toward advanced nanocarrier architectures designed to modulate drug release and enhance interfacial interactions with the ocular surface [16,17]. Various mucoadhesive delivery systems have been previously investigated for BT to overcome its rapid precorneal elimination, prolong its ocular retention, and enhance its ocular bioavailability. Eldeeb et al. [12] developed a BT proniosomal gel that significantly extended precorneal retention, achieving a five-fold increase in the ocular bioavailability. Moreover, Sathyavathi et al. [18] developed lyophilized nanovesicles incorporated into a carbopol-mucoadhesive system, providing sustained release and improved ocular bioavailability of BT. Additionally, Eudragit-based nanoparticles prepared via double emulsion–solvent evaporation have demonstrated the ability to prolong BT release, enhance intraocular pressure–lowering efficacy, and improve ocular tolerability [19]. Recently, hyaluronic acid-enriched cubosomal in situ gel for BT delivery was developed, providing a sol–gel transition upon ocular contact to enhance retention. This system demonstrated sustained release for 24 h, significantly improving bioavailability [20].

However, a critical innovation gap persists in the development of a delivery platform capable of actively mitigating rapid precorneal clearance via adhesion-based precorneal retention, while concurrently overcoming BT’s intrinsically poor encapsulation efficiency within lipid bilayers.

To address the existing therapeutic deficit, this research introduces rationally engineered, stearylamine-enriched liposomes, termed Aminosomes, designed to circumvent rapid precorneal clearance via electrostatic interfacial adhesion. By strategically integrating the cationic amphiphile stearylamine (SA) [21] into a lecithin-cholesterol scaffold, the platform generates a persistent surface electropositivity that facilitates high-affinity electrostatic coupling with the anionic sialic acid and sulfated residues of the ocular mucin glycocalyx [22]. This interfacial anchoring is hypothesized to establish a localized, bioadhesive drug reservoir that resists nasolacrimal washout, thereby maintaining a potent concentration gradient to drive sustained transcorneal flux [23,24,25]. Nevertheless, the stoichiometric incorporation of SA requires precise optimization to achieve the requisite surface potential for mucoadhesion while mitigating potential cytotoxic effects and maintaining the structural integrity of the lipid bilayer.

To mitigate the inherent challenges of low entrapment efficiency (EE) typically associated with loading of hydrophilic drugs like BT, the developmental framework employed a systematic multifactorial experimental design aimed at maximizing drug sequestration within the vesicular core. Given that BT preferentially partitions into the external aqueous phase [26], we modeled the synergistic interplay between three critical formulation parameters to optimize the thermodynamic stability of the payload within vesicles. Specifically, we evaluated: (i) phospholipid concentration, which governs bilayer lamellarity, membrane thickness, and the resultant internal aqueous volume; (ii) the initial drug payload, a primary determinant of the saturation kinetics during vesicle formation; and (iii) the lecithin-to-SA ratio, which modulates the surface charge density and packing parameters of the lipidic scaffold. By resolving the complex interactions between these variables, this study sought to engineer a nanoplatform that balances high loading capacity with robust mucoadhesive anchoring, thereby transforming the pharmacokinetic profile of BT from a fluctuating bolus delivery to a sustained therapeutic flux, thereby mitigating local toxicity and enhancing the long-term management of intraocular pressure.

Prior strategies have addressed the encapsulation deficits of BT through the formulation of gelatinized-core liposomes using conventional thin-film hydration [27]. This research explores the optimization of ethanol injection technique parameters as a superior alternative. The ethanol injection technique was strategically selected for its straightforward methodology, minimal residual organic solvent profile, and inherent industrial scalability, alongside its capacity to generate vesicles with a high degree of monodispersity [28]. To circumvent the tendency of the hydrophilic BT to partition into the bulk aqueous phase in the ethanol injection technique, we systematically investigated a suite of formulation variables hypothesized to favor solute sequestration. Building on evidence that EE in the ethanol injection can be augmented by increasing phospholipid concentration and optimizing the volumetric ratio of the aqueous phase [29]. Our approach further examined the impact of phase-specific drug incorporation by modulating the initial payload and introducing the drug within the ethanolic phase. We aimed to influence the nucleation kinetics of the Aminosomes to stabilize the drug within the internal aqueous compartment, thereby achieving a high-capacity reservoir for sustained ocular delivery.

Consequently, the objectives of this investigation were delineated to evaluate the ethanol injection technique as a robust fabrication strategy for BT-loaded Aminosomes, specifically interrogating the formulation parameters that mitigate the thermodynamic drive for hydrophilic drug partitioning to maximize entrapment efficiency. Through a systematic optimization of the vesicles’ physicochemical architecture, we sought to harmonize nanometric dimensions with a high-density mucoadhesive surface charge. Ultimately, this research aimed to validate the therapeutic superiority of the optimized cationic nanoplatform through in vivo pharmacodynamic assessment, establishing its potential to enhance ocular bioavailability and provide a sustained, long-term pharmacological intervention for the management of glaucoma.

## 2. Materials and Methods

### 2.1. Materials

Brimonidine Tartrate (BT) was kindly obtained as a gift from EVA Pharmaceutics (Cairo, Egypt). Absolute ethanol and stearylamine (SA) were acquired from Piochem Chemical Co. (Cairo, Egypt). Cholesterol was purchased from Advent ChemBio Pvt Ltd. (Mumbai, India). Egg yolk lecithin was purchased from Sigma Aldrich Chemical Co. (St. Louis, MO, USA). All other used chemicals were of analytical grade.

### 2.2. Animals

Six New Zealand male albino rabbits (2.00–3.00 kg) were purchased from the animal house at the Faculty of Pharmacy, Cairo University, to be included in the in vivo studies. The rabbits were placed individually in cages (one per cage) and supplied with standard food and water for seven days before carrying out the experiments in order to accommodate them. Visual examination of the rabbit eyes was performed before the experiment by a slit of light to detect any sign of irritation, redness, or inflammation. Rabbits with any unusual eye signs were excluded from the study.

### 2.3. Methods

#### 2.3.1. Preparation of BT-Loaded Aminosomes

BT-loaded Aminosomes (AMS) were prepared using the ethanol injection technique. Precisely weighed quantities of egg yolk lecithin, cholesterol, and stearylamine (SA), together with BT, were dissolved in ethanol under gentle heating to ensure complete solubilization of all components. Concurrently, distilled water was preheated to 60 ± 2 °C to facilitate rapid lipid self-assembly upon injection. The ethanolic lipid–drug solution was then injected dropwise into the preheated aqueous phase under continuous magnetic stirring at 1000 rpm (Wise Stir, Daihan Scientific Co., Ltd., Wonju-si, Gangwon-do, Republic of Korea). Upon contact with the aqueous medium, spontaneous lipid self-organization occurred, resulting in the formation of stearylamine-enriched liposomal vesicles (Aminosomes). The dispersion was maintained at 60 ± 2 °C and stirred at 1000 rpm for 60 min to allow complete evaporation of ethanol [30,31]. Following solvent removal, the resultant dispersion was sonicated using a bath sonicator (Crest Ultrasonics Corp., Trenton, NJ, USA) to disrupt any lipid aggregates and promote uniform vesicle size distribution. The prepared AMS were subsequently transferred to amber glass vials and stored overnight at 4 °C to allow vesicular stabilization prior to further characterization and evaluation [2,32].

#### 2.3.2. A 3^2^ × 2^1^ Full Factorial Design Analysis for BT-Loaded Aminosomes

Preliminary screening studies were executed to explore the influence of formulation and process variables on the physicochemical attributes of the engineered AMS. The investigated parameters included the loading of BT, lecithin, cholesterol, and SA, alongside sonication duration and phase volume ratios. Based on these initial outcomes, specific conditions, including a cholesterol content of 20 mg, a sonication duration of 10 min, and an ethanolic-to-aqueous phase ratio of 4:10 *v*/*v*, were selected, as they provided the best results for achieving the required vesicular characteristics.

Conversely, the concentrations of BT, lecithin, and SA were identified as critical material attributes (CMAs) significantly governing vesicular properties, thereby necessitating rigorous optimization via a systematic experimental design.

A 3^2^ × 2^1^ factorial matrix was constructed using Design-Expert^®^ 13 software (Stat-Ease, Inc., Minneapolis, MN, USA), where lecithin concentration (X_1_) and the lecithin-to-SA ratio (X_2_) were varied across three levels, while the BT payload (X_3_) was evaluated at two levels [33]. The investigated levels for each factor are detailed in Table 1. This multivariate approach was utilized to systematically deconvolute the main and interaction effects of the independent variables on the critical quality attributes (CQAs): PS (Y_1_), PDI (Y_2_), ZP (Y_3_), and EE (Y_4_). Based on the design matrix, eighteen distinct AMS formulations were synthesized as outlined in Table 2. All formulations were manufactured in triplicate, with data reported as mean ± standard deviation (SD). Statistical validation was performed via Analysis of Variance (ANOVA) using Design-Expert^®^, with significance established at *p* < 0.05.

#### 2.3.3. In Vitro Characterizations of BT-Loaded Aminosomes

##### PS, PDI, and ZP Measurements

PS, PDI, and ZP of the eighteen factorial formulations were determined using a Zetasizer Nano ZS (Malvern Instruments, Malvern, UK). The PS and PDI were assessed via Dynamic Light Scattering (DLS) [34], while ZP was quantified using Electrophoretic Light Scattering (ELS) to determine surface charge [35]. Prior to analysis, samples were diluted 1:20 (*v*/*v*) with deionized water to mitigate multiple scattering effects and ensure appropriate count rates. All measurements were conducted at a controlled temperature of 25 ± 2 °C [36,37]. Analysis was performed in triplicate, with results reported as the mean of independent measurements [38,39,40].

##### Drug Content and Entrapment Efficiency Measurement

Total drug content was quantified by lysing a 0.1 mL aliquot of the AMS dispersion in 10 mL of ethanol, ensuring complete vesicular disruption and solubilization of the entrapped BT [41]. The solution was analyzed spectrophotometrically (Shimadzu UV-1601 PC, Kyoto, Japan) at a λ_max_ of 257.8 nm. To determine the entrapment efficiency (EE%), the formulations were fractionated to separate unentrapped (free) BT from the vesicular phase. A 1.5 mL sample was subjected to cooling ultracentrifugation at 15,000 rpm and 4 °C for 1 h (Beckman, Fullerton, Canada) [42]. The resulting supernatant, containing the free drug, was isolated and quantified via UV spectrophotometry at 257.8 nm [12]. The EE% was subsequently calculated using the following equation [43,44]:

(1) $$\mathrm{EE}\%\;=\;\frac{Total\;BT\;concentration\left(mg/mL\right)-Free\;BT\;concentration\left(mg/mL\right)}{Total\;BT\;concentration\;(mg/mL)}\times \;100$$

#### 2.3.4. Statistical Optimization to Elucidate the Optimized BT-Loaded Aminosomes

Following the physicochemical characterization of the experimental formulations, multi-criteria optimization was performed using a desirability function approach, with constraints established for each response (Table 1) to identify the optimal processing window aligned with specific therapeutic targets. Specifically, PS (Y_1_) and PDI (Y_2_) were minimized to ensure a monodisperse, nanometric population capable of facilitating transcorneal permeation while avoiding ocular irritation. Conversely, ZP (Y_3_) was maximized to enhance electrostatic coupling with the anionic ocular mucin, thereby establishing a mucoadhesive drug reservoir, while EE (Y_4_) was maximized to ensure a high-capacity payload for sustained delivery. From the generated solutions, the formulation exhibiting the highest composite desirability index was selected as the optimized AMS candidate, representing the ideal balance between nanometric architecture, robust drug sequestration, and mucoadhesion potential. The optimized formulation was subsequently prepared independently and subjected to in vitro characterization. The experimentally obtained results were then compared with the software-predicted values, according to the following equation, to verify the accuracy of the optimization process.

% Deviation = (|Predicted value − Observed value|/(Observed value)) × 100(2)

#### 2.3.5. Further Characterizations of the Optimized BT-Loaded Aminosomes

##### Transmission Electron Microscope (TEM)

Morphology of the optimized AMS was elucidated using Transmission Electron Microscopy (TEM; Jeol JEM1230, Tokyo, Japan). Samples were prepared via a negative staining technique to enhance contrast. Briefly, a droplet of the vesicular dispersion was deposited onto a carbon-coated copper grid and allowed to adsorb. Subsequently, the sample was stained with 1% (*w*/*v*) sodium phosphotungstate [45]. Excess staining reagent was wicked away using absorbent filter paper, and the grid was air-dried at ambient temperature for 30 min prior to imaging [46].

##### In Vitro Release Study of BT from the Optimized Aminosomes

The in vitro release kinetics of BT from the optimized AMS, relative to a free BT solution, were assessed using the dialysis bag diffusion technique. A Spectra/Por^®^ semi-permeable membrane (MWCO 12–14 kDa) was employed to permit the diffusion of free drug while retaining the vesicular carriers. Dialysis sacs were charged with 2 mL of either the optimized AMS dispersion or the BT solution (equivalent to a 2 mg BT payload) and immersed in a receiver compartment containing 100 mL of phosphate buffer (pH 7.4). The system was maintained at 37 ± 0.5 °C under constant agitation (100 rpm) in a shaking water bath (GFL, Gesellschatt Laboratories, Berlin, Germany) to simulate physiological conditions and minimize boundary layer resistance [47]. At predetermined time intervals (5, 10, 15, 30, and 45 min; 1, 2, 3, 4, 6, 8, and 12 h), 2 mL samples were withdrawn from the receiver medium and immediately replaced with an equal volume of fresh buffer to maintain sink conditions. The concentration of released BT was quantified spectrophotometrically as described previously. Cumulative drug release percentages were calculated according to the following equation:

(3) $$Qn=\frac{CnxVr+\;\sum_{i=1}^{n-1}CixVs}{initial\;drug\;content}\times 100$$

where

Qn: cumulative percentage of drug released at the nth sampling time.

Cn: drug concentration in the receiver medium at the nth sampling time.

Vr: total volume of the receiver medium.

Vs: volume of each sample withdrawn for analysis.

$\sum_{i=1}^{n-1}Ci$: sum of the drug concentrations measured in all previously withdrawn samples.

To elucidate the underlying mechanism governing drug release, the data were mathematically fitted to various kinetic models: Zero-order (concentration-independent), First-order (concentration-dependent), Higuchi (diffusion-controlled), Hixson–Crowell (erosion-controlled), and Korsmeyer–Peppas (mixed-mechanism). The goodness of fit for each model was evaluated via the coefficient of determination (R^2^) to identify the predominant release mechanism.

##### Mucoadhesion Assessment

The mucoadhesive potential of the optimized AMS formulation was assessed via an in vitro mucin interaction assay, quantifying the electrostatic neutralization of the cationic vesicles by anionic mucin glycoproteins. A dispersion of porcine stomach mucin (type II) was prepared in distilled water (1% *w*/*v*) and allowed to hydrate completely. The mucin solution was added dropwise to an equal volume of the optimized AMS dispersion. Stirring was continued for an additional 5 min, after which the mixture was allowed to equilibrate overnight at room temperature [48]. Prior to incubation, the ZP of the mucin solution and the optimized AMS dispersion were analyzed using a Zetasizer (Malvern Instruments, Malvern, Worcestershire, UK) and compared to the ZP of the AMS–mucin complex. The degree of mucoadhesion was inferred from the magnitude of the zeta potential shift. A significant reduction in the net positive charge serves as a mechanistic indicator of strong adhesive interaction, driven by the electrostatic attraction between the cationic SA residues of the AMS and the negatively charged sialic acid and sulfate moieties of the mucin [49].

##### Fourier Transform Infrared (FT-IR) Analysis

To ensure the physicochemical stability of the vesicular structure during sublimation, mannitol (2% *w*/*v*) was incorporated into the optimized AMS dispersion as a cryoprotectant prior to lyophilization (Lyovapor L-200, Büchi Labortechnik AG, Flawil, Switzerland). For Fourier Transform Infrared (FT-IR) analysis, the lyophilized product was finely triturated and geometrically mixed with spectroscopic-grade potassium bromide (KBr). This mixture was compressed under high pressure to form translucent pellets. A reference spectrum of pure BT was acquired under identical conditions. Scans were executed over the wavenumber range of 4000–400 cm^−1^ using a Shimadzu IR-436 spectrometer (Shimadzu Corp., Kyoto, Japan). The resulting spectra were scrutinized for shifts in characteristic absorption bands or the appearance of new peaks, which would indicate intermolecular interactions or alterations in the drug’s chemical environment.

##### X-Ray Diffraction Analysis

The solid-state physical characteristics of pure BT and the lyophilized optimized AMS formulation were investigated using X-ray powder diffraction (XRPD) to assess crystallinity and evaluate the potential amorphization of the drug within the lipid matrix. Lyophilized samples were prepared as previously described. The powders were gently packed into sample holders to obtain a flat, uniform surface and minimize preferred orientation effects. Diffraction patterns were acquired using an X-ray diffractometer equipped with a Cu-Kα radiation source (λ = 1.5406 Å), operated at an accelerating voltage of 40 kV and a current of 30 mA. Scans were performed over a 2θ range of 10–80° with a step size of 0.02° and a scanning rate of 2° (2θ) per minute under ambient conditions. The resulting diffractograms were analyzed for the presence or attenuation of characteristic Bragg reflections.

#### 2.3.6. In Vivo Evaluations of the Optimized BT-Loaded Aminosomes Formulation

In vivo experimental procedures were conducted in accordance with the ARRIVE 2.0 guidelines and approved by the Research Ethics Committee of the Faculty of Pharmacy, Cairo University, Egypt (Approval No: PI 3549). All protocols adhered to the ethical standards for animal care outlined in the “U.S. National Institutes of Health’s Guide for the Care and Use of Laboratory Animals” (NIH Publication No. 85–23, revised 2011) to ensure the highest standards of animal welfare.

##### Pharmacodynamic Evaluation of the IOP-Reducing Effect of the Optimized BT-Loaded Aminosomes

Prior to the study, rabbits were acclimatized to a controlled 12 h light/12 h dark cycle to synchronize circadian rhythms, thereby stabilizing baseline IOP fluctuations and ensuring accurate pharmacodynamic assessment. A single-dose, two-period crossover design was employed with a one-week washout interval to eliminate carryover effects [50]. Six New Zealand albino rabbits (2.0–3.0 kg) were randomized into two groups (A and B; *n* = 3) [51]. Baseline IOP (IOP_t=0_) was quantified for all subjects using a calibrated tonometer (SchiÖtz Tonometer Rudolf Riester GmbH and Co. KG, Jungingen, Germany) [52]. Subsequently, 20 μL of the optimized AMS formulation was instilled into the lower conjunctival sac of the right eye for Group A, while Group B received an equal dose of the reference BT solution. The contralateral eye in both groups received 20 μL of sterile 0.9% (*w*/*v*) NaCl solution to serve as a negative control [8]. IOP measurements were acquired in triplicate at predetermined intervals (0.25, 0.5, 1, 1.5, 2, 3, 5, 7, 9, and 24 h post-instillation). Data are reported as means ± SD, with 95% confidence intervals calculated to provide a measure of variability. The pharmacodynamic efficacy was quantified as the percentage reduction in IOP (IOP_red%_), calculated via the subsequent formula [16,53]:

(4) $$\mathrm{The}\;\%\;\mathrm{reduction}\;\mathrm{in}\;\mathrm{IOP}\;=\;\frac{IOP\;at\;control\;eye-IOP\;at\;treated\;eye}{IOP\;at\;control\;eye}\times \;100$$

Non-compartmental analysis was conducted using Phoenix WinNonlin software (Version 8.3, Certara, Princeton, NJ, USA) to derive key bioavailability parameters [1,52]:

- **E_max_:** Maximum percentage reduction in IOP.
- **T_max_:** Time to reach maximal effect.
- **MRT:** Mean residence time of the therapeutic effect.
- **T_1/2_:** Biological half-life of the IOP reduction.
- **AUC_0–24h_:** Area under the effect-time curve, representing total therapeutic exposure [54].

Statistical comparisons between the optimized AMS formulation and reference solution were performed using IBM SPSS Statistics version 19.0 (IBM Corp., Armonk, NY, USA) (Student’s *t*-test, *p* < 0.05) [1,8].

##### Assessment of the Ocular Irritation by the Draize Test

To evaluate the local safety profile and biocompatibility of the optimized AMS formulation upon topical administration, an ocular irritation study was conducted using six healthy male albino rabbits (2.0–3.0 kg). Following a seven-day acclimatization period with ad libitum access to standard diet and water, a rigorous baseline ophthalmic examination was performed to exclude subjects exhibiting pre-existing ocular pathology, such as conjunctival hyperemia, chemosis, or epiphora [52]. The qualified animals were randomized into two cohorts (*n* = 3), wherein Group A received 20 μL of the optimized AMS formulation and Group B received an equimolar dose of the reference BT solution instilled into the lower conjunctival sac of the right eye; simultaneously, the left eye served as a negative control treated with 20 μL of sterile 0.9% (*w*/*v*) NaCl solution [55]. Ocular tissue integrity was subsequently assessed via macroscopic inspection under focal illumination at predetermined intervals (0, 0.5, 1, 2, 3, 5, 7, 9, and 24 h post-instillation), and the degree of irritation was quantified using the standardized Draize scale, ranging from 0 (no irritation) to +3 (severe erythema and edema) [56,57].

##### Histopathological Assessment

To comprehensively evaluate the ultrastructural integrity and cellular architecture of ocular tissues following repeated administration, a histopathological assessment was conducted on six rabbits randomized into two cohorts (*n* = 3) [52]. To simulate sub-chronic exposure stress, the right eye of each subject was subjected to an intensive dosing regimen, receiving 100 µL of either the optimized AMS formulation (Group A) or the reference BT solution (Group B) three times daily for seven consecutive days, while the contralateral eye received sterile 0.9% (*w*/*v*) NaCl solution as a negative control [58]. Following the treatment period, animals were euthanized, and corneas were surgically excised and immediately fixed in 10% (*v*/*v*) neutral buffered formalin to prevent autolysis and preserve tissue morphology. The fixed tissues subsequently underwent graded dehydration and paraffin embedding [59] to generate solid blocks. Finally, serial sections were prepared, stained with Hematoxylin and Eosin, and scrutinized for pathological alterations using a digital microscope (DMS1000 B; Leica, Cambridge, UK) [60].

## 3. Results and Discussion

### 3.1. A 3^2^ × 2^1^ Full Factorial Design Analysis for BT-Loaded Aminosomes

The in vitro characterization datasets derived from the eighteen AMS formulations, constructed via the 3^2^ × 2^1^ factorial design (Table 2), were subjected to statistical analysis using Design-Expert^®^ software (version 13). Table 3 delineates the fit statistics for the optimized models. Notably, all response variables exhibited adequate precision values surpassing the critical threshold of 4. This metric indicates a robust signal-to-noise ratio, thereby substantiating the experimental design’s resolution and its efficacy in interrogating the defined design space [61]. Furthermore, the discrepancy between the predicted R^2^ and adjusted R^2^ values was consistently maintained below 0.2 across all responses. This convergence corroborates the statistical robustness of the final models, confirming their capability to accurately fit the experimental data and reliably predict response trajectories as a function of the independent variables [62].

Regression modeling yielded the following mathematical expressions characterizing the response surfaces for PS, PDI, ZP, and EE:

PS = 255.69 + 100.07A − 12.60B + 23.20C − 2.80AB + 50.33AC + 12.35BC

PDI = 0.2934 + 0.0786A − 0.0334B − 0.0001C − 0.0787AB + 0.0117AC − 0.0333BC

ZP = 22.31 + 4.57A − 10.14B + 5.43C + 2.88AB − 6.04AC − 1.90BC + 15.66A^2^ + 4.42B^2^ + 0.00C^2^

EE = 43.61 + 6.30A + 1.25B + 4.28C + 1.84AB − 2.47AC − 2.25BC + 5.08A^2^ + 0.18B^2^ + 0.00C^2^

where A, B, and C are coded variables for lecithin concentration (X_1_), lecithin to SA ratio (X_2_), and BT amount (X_3_), respectively.

#### 3.1.1. PS, PDI, and ZP Measurements

PS is a pivotal determinant in ocular drug delivery, as it directly influences precorneal residence, corneal permeation, and overall therapeutic efficiency. Nanosized systems (typically <300 nm) exhibit enhanced retention within the tear film, reduced irritation, and improved penetration across the corneal epithelium via paracellular and transcellular pathways. In contrast, larger particles are more susceptible to rapid elimination through blinking and nasolacrimal drainage, limiting drug absorption [63]. Moreover, PS governs surface area-to-volume ratio, thereby modulating drug release kinetics where smaller vesicles generally enable faster and more uniform release, while larger ones may exhibit slower, diffusion-limited profiles.

As delineated in Table 2, the developed AMS formulations exhibited PS ranging from 144.6 ± 1.69 nm to 482.95 ± 9.86 nm. Notably, thirteen of the formulations fell within the specific sub-300 nm window predictive of enhanced ocular retention.

**Figure 1:**
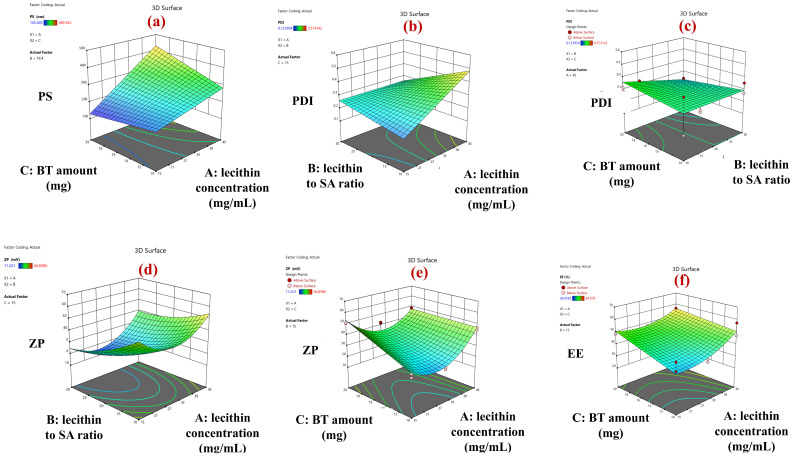
Response surface plots showing effects of (**a**) lecithin concentration and BT amount on PS, (**b**) lecithin concentration and lecithin to SA ratio on PDI, (**c**) lecithin to SA ratio and BT amount on PDI, (**d**) lecithin concentration and lecithin to SA ratio on ZP, (**e**) lecithin concentration and BT amount on ZP, and (**f**) lecithin concentration and BT amount on EE%.

Statistical interrogation via ANOVA identified lecithin concentration (X_1_) as a significant determinant of PS (*p* < 0.0001), as illustrated in Figure 1a. A positive concentration-dependent correlation between lipid load and vesicular size was observed, where formulations comprising 45 mg/mL of lecithin displayed larger mean diameters (366.92 ± 4.11 nm) compared to those utilizing 30 mg/mL (240.60 ± 2.98 nm) and 15 mg/mL (165.84 ± 2.81 nm). These data corroborate previous reports demonstrating vesicle enlargement with increasing lecithin concentration [64]. Mechanistically, this phenomenon is attributed to the self-assembly dynamics of amphiphilic lipids. At higher lecithin concentrations, increased availability of bilayer-forming material promotes the nucleation of multilamellar vesicles (MLVs) with expanded aqueous cores and thicker lipid shells, resulting in larger hydrodynamic diameters [65]. The observed increase in PS at higher lecithin concentrations can be further elucidated by the molecular composition of the lipid source. Egg yolk lecithin is a natural mixture containing both Phosphatidylcholine (PC) and Phosphatidylethanolamine (PE). At the higher lecithin concentration, the increased presence of the cone-shaped PE molecules likely induces significant curvature stress within the bilayer. To minimize the free energy associated with this stress, the system favors the formation of larger vesicles with lower mean curvature [66]. Furthermore, it is hypothesized that increased lipid concentrations modulate the rheological properties of the dispersion, leading to a concentration-dependent rise in viscosity. Such elevated viscosity likely raises the threshold for acoustic cavitation during sonication, impeding the shear forces required to disrupt aggregates, consequently resulting in the persistence of larger vesicular structures [67]. In the absence of direct rheological measurements, the viscosity-mediated mechanism is regarded as a plausible interpretation rather than an experimentally validated one.

BT amount exerted a statistically significant effect on the PS of the developed AMS (*p* = 0.0374). Notably, a significant interaction between lecithin concentration (X_1_) and BT amount (X_3_) was observed (*p* = 0.0005), indicating that the influence of BT load on PS was strongly dependent on the phospholipid content. As depicted in the response surface plot (Figure 1a), increasing BT loading resulted in a more pronounced enlargement of vesicle size at higher lecithin concentrations, whereas the effect was comparatively attenuated at lower lipid levels. This behavior can be rationalized by the increased availability of phospholipid bilayer material at elevated lecithin concentrations, which facilitates greater drug accommodation within the vesicular system, promoting bilayer expansion and vesicle swelling rather than drug expulsion. In contrast, at lower lecithin concentrations, limited bilayer capacity restricts BT incorporation, thereby moderating vesicle growth. Collectively, these findings underscore the synergistic interplay between drug loading and lipid concentration in governing AMS size and highlight the necessity of balancing these parameters to achieve optimal nanoscale dimensions.

The polydispersity index (PDI) serves as a quantitative metric for colloidal heterogeneity, reflecting the breadth of the particle size distribution arising from inherent synthesis variability or aggregation phenomena. A PDI value < 0.3 is generally accepted as the threshold for monodispersity in lipid-based nanocarriers [68]. PDI is a critical quality attribute in ocular drug delivery systems, as it reflects the uniformity of vesicle size distribution, which directly governs formulation performance. Low PDI values indicate a homogeneous population, ensuring predictable drug release kinetics, reproducible corneal permeation, and consistent precorneal retention. In contrast, elevated PDI denotes heterogeneity in vesicle size and structure, which can lead to variable release behavior, inconsistent mucoadhesion, and non-uniform ocular bioavailability. Therefore, maintaining a narrow size distribution is essential for achieving controlled therapeutic outcomes and minimizing variability in ocular drug delivery systems.

The factorial design AMS formulations exhibited PDI values ranging from 0.16 ± 0.00 to 0.56 ± 0.02. Notably, twelve formulations achieved PDI values ≤ 0.3, signifying a robust capacity of the design space to yield highly uniform vesicular populations. ANOVA revealed that PDI was significantly modulated by both lecithin concentration (X_1_) (*p* < 0.0001) and lecithin to SA ratio (X_2_) (*p* < 0.0062).

Consistent with the PS data, an elevation in lecithin concentration precipitated a marked increase in PDI (Figure 1b), where formulations comprising 45 mg/mL of lecithin exhibited higher heterogeneity (PDI of 0.39 ± 0.05) compared to those with 30 mg/mL (0.28 ± 0.05) and 15 mg/mL (0.215 ± 0.01). The elevated PDI at high lecithin concentrations can be attributed to the thermodynamic drive toward multilamellarity. The increased lipid-to-water ratio favors the formation of concentric bilayer structures with varying lamellar counts, inherently broadening the size distribution [65]. Furthermore, the reduced inter-vesicular distance in high-lipid dispersions increases the probability of spontaneous vesicle fusion, leading to a heterogeneous population of original and coalesced vesicle [69]. Moreover, this increased PDI can be attributed to the probable rheological consequences of high lipid loading. Elevated lecithin concentrations likely increase the viscosity, which dampens the propagation of acoustic waves during sonication. This attenuation of cavitational shear forces impairs the de-aggregation process, resulting in a broader size distribution and elevated PDI [70].

The homogeneity of the dispersion was inversely correlated with the concentration of SA, where formulations utilizing lower lecithin: SA ratios (10:1 and 15:1), which correspond to higher absolute SA loads, displayed higher PDI values compared to the 30:1 ratio (Figure 1b).

The observed increase in PDI at higher SA concentrations can be attributed to the molecular orientation and packing constraints of the cationic lipid within the lecithin bilayer. SA intercalates its C18 alkyl chain among the phospholipid acyl chains, while its protonated amine headgroup projects into the aqueous interface. At elevated SA loads, a volumetric mismatch arises between the bulky, repulsive amine headgroups and the relatively compact acyl chains, disrupting the bilayer’s Critical Packing Parameter (CPP) and inducing curvature instability [71]. This discrepancy likely introduces packing defects within the phospholipid matrix, where the intercalation of excessive SA molecules perturbs the ordered arrangement of the acyl chains. Such membrane destabilization compromises the uniformity of vesicle formation during the self-assembly process, ultimately resulting in a more heterogeneous population and the recorded elevation in PDI.

It is worth noting that the effect of lecithin to SA ratio on PDI was significantly modulated by two interaction effects, specifically with lecithin concentration (*p* < 0.0001), and BT amount (*p* = 0.0062).

As illustrated in Figure 1b, the deleterious impact of increasing lecithin concentration on PDI was pronounced solely in formulations utilizing higher SA loads (ratios 10:1 and 15:1); conversely, this lipid-driven heterogeneity was effectively mitigated at the 30:1 ratio, suggesting that minimizing SA stabilizes the bilayer against lipid-induced aggregation.

Furthermore, the ability of the 30:1 lecithin-to-SA ratio to minimize polydispersity was strongly dependent on the drug payload, achieving a statistically significant reduction in PDI only when combined with 20 mg of BT. In contrast, at a lower BT load (10 mg), variations in the lecithin-to-SA ratio did not result in a significant change in PDI (Figure 1c). This conditional effect underscores a nuanced physicochemical interplay, wherein an optimal drug–lipid stoichiometry likely promotes more ordered bilayer packing and reduces inter-vesicular variability, ultimately enhancing colloidal homogeneity.

Zeta potential (ZP), defined as the electrokinetic potential difference between the dispersion medium and the stationary fluid layer attached to the dispersed particle [72], governs two critical performance parameters of the developed BT-loaded AMS. First, ZP serves as an indicator of colloidal stability; values exceeding ± 30 mV provide sufficient electrostatic repulsion to prevent vesicular aggregation and maintain monodispersity [68]. Second, ZP is a primary determinant of in vivo ocular residence. The corneal surface and tear film mucins are decorated with sialic acid and sulfate residues, generating a net negative physiological charge [73]. Consequently, engineering vesicles with a positive surface charge facilitates electrostatic adhesion to the ocular mucosa. This mucoadhesive interaction prolongs precorneal retention, thereby enhancing the transcorneal concentration gradient and subsequent intraocular absorption, a mechanism pivotal for augmenting and sustaining the intraocular pressure (IOP)-reducing efficacy of BT [74].

The developed AMS formulations exhibited ZP values ranging from 11.3 ± 0.14 mV to 63.95 ± 4.17 mV. Notably, twelve formulations surpassed the +30 mV stability threshold, confirming robust electrostatic stabilization. The universal positivity across all formulations is attributed to the incorporation of SA, an amphiphile presenting a primary amine group that undergoes protonation in the aqueous phase [75].

ANOVA identified significant main effects for all three independent variables on ZP: lecithin concentration (X_1_, *p* = 0.0003), lecithin to SA ratio (X_2_, *p* < 0.0001), and BT amount (X_3_, *p* < 0.0001) (Figure 1d,e).

The influence of lecithin content on ZP displayed a non-linear profile. Formulations with 15 mg/mL lecithin yielded a mean ZP of 38.88 ± 1.74 mV. While an increase to 30 mg/mL significantly attenuated the charge to 27.31 ± 0.52 mV, a further increase to 45 mg/mL unexpectedly restored the high positive charge (47.06 ± 1.52 mV) (Figure 1d).

As anticipated, surface charge magnitude was positively correlated with the molar fraction of the cationic lipid SA. Formulations with the lowest SA content (lecithin to SA ratio of 30:1) exhibited the lowest mean ZP (27.02 ± 0.36 mV). Increasing the SA load via the 15:1 and 10:1 ratios resulted in a progressive elevation of ZP to 38.92 ± 0.93 mV and 47.31 ± 2.5 mV, respectively (Figure 1d). This trend substantiates a concentration-dependent augmentation of surface cationic character, attributable to increased exposure of protonated amine functionalities at the vesicle interface.

Increasing BT payload from 10 mg to 20 mg elicited a significant rise in mean ZP from 32.00 ± 1.20 mV to 43.51 ± 1.32 mV (Figure 1e). This increment in surface charge density may be attributed to two synergistic mechanistic pathways. First, the partial adsorption or interfacial partitioning of protonated BT molecules at the outer bilayer surface likely contributes to the electrokinetic potential, a phenomenon previously documented for cationic drugs influencing liposomal surface charge [76]. Second, an increased drug concentration within the aqueous Stern layer surrounding the lipid headgroups may further modulate the charge density at the shear plane. Definitive validation of the molecular localization of BT within the lipid matrix would require advanced biophysical techniques, such as Differential Scanning Calorimetry (DSC) to detect changes in phase transition temperature, or solid-state NMR and Small-Angle X-ray Scattering (SAXS) to probe drug–headgroup spatial proximity and lamellar repeat spacing.

A statistically significant interaction was detected between lecithin concentration (X_1_) and BT content (X_3_) (*p* < 0.0001) (Figure 1e), indicating that the influence of BT on surface charge was contingent upon the lipid concentration. Specifically, BT-mediated elevation of ZP was pronounced at lower lecithin levels (15 and 30 mg/mL), whereas this effect was effectively abolished in formulations containing the highest lecithin load (45 mg/mL). This behavior suggests that at elevated phospholipid concentrations, the abundance of anionic phosphate moieties within the phosphatidylcholine headgroups exerts a charge-shielding effect, counterbalancing the cationic contribution imparted by BT molecules and thereby attenuating the net increase in surface positive charge.

#### 3.1.2. Entrapment Efficiency Measurement

Optimizing EE is crucial for the ocular delivery of BT, a hydrophilic drug susceptible to rapid precorneal clearance. High EE within the lipid-based AMS system effectively sequesters the drug from the tear film, minimizing premature washout. Furthermore, by facilitating interactions with the lipophilic corneal epithelium, high EE enhances drug permeation and penetration into the aqueous humor. This also ensures a sustained release of BT, improving bioavailability and reducing the need for frequent dosing [77]. However, the intrinsic hydrophilicity of BT (aqueous solubility around 34 mg/mL) poses a thermodynamic challenge for vesicular encapsulation, often resulting in relatively low drug loading [78]. To circumvent this limitation, we employed a modified ethanol injection technique wherein BT was solubilized within the organic phase, facilitating its intercalation during the initial vesicle self-assembly. Furthermore, the design space interrogated the impact of lecithin mass, BT payload, and SA ratios on encapsulation metrics.

The developed AMS formulations yielded EE values ranging from 28.70 ± 0.91% to 60.30 ± 5.34%. Notably, seven formulations surpassed the 50% threshold, validating the utility of the ethanol injection protocol in overcoming the leakage propensity of water-soluble cargoes.

Statistical analysis via ANOVA identified significant main effects for both X_1_: lecithin concentration (*p* < 0.0001) and X_3_: BT amount (*p* = 0.0001) on EE%.

A distinct positive correlation was observed between lipid mass and encapsulation efficiency (Figure 1f). While formulations containing 15 mg and 30 mg/mL of lecithin exhibited comparable mean EE% values (42.61 ± 2.09% and 43.53 ± 3.16%, respectively), increasing the lipid load to 45 mg/mL significantly elevated the mean EE% to 54.61 ± 2.44%. Mechanistically, this enhancement is closely linked to the vesicular hypertrophy observed in the particle size analysis. The formation of larger vesicles at higher lipid concentrations results in a geometric expansion of the internal aqueous core volume, maximizing the reservoir capacity available for sequestering the hydrophilic drug solution, a phenomenon well-documented in liposomal formulations [79].

Increasing the initial BT input from 10 mg to 20 mg precipitated a significant rise in mean EE% from 42.27 ± 2.68% to 51.57 ± 2.45% (Figure 1f). This improvement is likely governed by mass transport dynamics; a higher initial drug concentration amplifies the chemical potential gradient across the forming bilayer interface. This intensified driving force accelerates the passive diffusion of BT into the intraliposomal compartment during vesiculation, thereby shifting the equilibrium toward higher encapsulation before the bilayer creates a complete barrier [80].

### 3.2. Elucidation of the Optimized BT-Loaded Aminosomes Formulation

To identify the optimal AMS formulation coordinates within the defined experimental space, numerical optimization was executed via Design-Expert^®^ software (version 13). Formulation F10 (comprising 15 mg/mL lecithin, a 10:1 Lecithin: SA ratio, and 20 mg BT) was identified as the lead candidate as it exhibited the highest desirability score (0.858). The optimized aminosomal formulation demonstrated physicochemical characteristics that are well-aligned with the requirements for effective ocular drug delivery. The observed particle size (153.4 nm) falls within the optimal nanoscale range for reduced ocular irritation, enhanced corneal permeation and prolonged precorneal retention, while the low PDI (0.22) indicates a relatively uniform vesicular population, ensuring reproducible performance. The high positive zeta potential (+63.95 mV) is particularly advantageous, as it promotes electrostatic interaction with the negatively charged ocular surface, thereby enhancing mucoadhesion and residence time. Additionally, the satisfactory entrapment efficiency (60.3%) supports adequate drug loading and sustained release behavior. As delineated in Table 4, a comparative analysis between the experimentally measured in vitro characteristics of F10 and the software-predicted values revealed acceptable congruence. This predictive accuracy substantiates the robustness of the adopted regression models and confirms the reliability of the multilevel factorial design in navigating the complex physicochemical landscape of AMS synthesis.

### 3.3. Transmission Electron Microscopy

**Figure 2:**
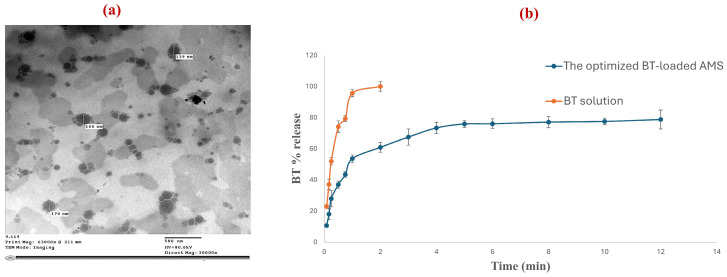
(**a**) TEM micrograph of the optimized AMS, (**b**) release profile of BT from the optimized AMS compared to BT solution.

The ultrastructural morphology of the optimized AMS formulation was elucidated via transmission electron microscopy (TEM), as depicted in Figure 2a. The TEM micrographs revealed well-defined, discrete vesicles exhibiting a characteristic spherical morphology. The observed vesicular diameters were highly congruent with the hydrodynamic size distribution previously obtained via dynamic light scattering (DLS) using the Malvern Zetasizer [81]. Furthermore, the images confirmed a high degree of monodispersity, characterized by an absence of vesicular coalescence or significant aggregation. This morphological integrity substantiates the efficacy of the ethanol injection technique and underscores the robust colloidal stability of the nanocarriers, likely facilitated by the high ZP, which prevents inter-vesicular proximity and subsequent fusion.

### 3.4. In Vitro Release Study and Kinetics Analysis

The release profiles of BT from the optimized AMS formulation and the reference BT aqueous solution are delineated in Figure 2b. The liberation of BT from the AMS exhibited a distinct biphasic kinetic trajectory, characterized by an initial rapid-release phase followed by a prolonged, controlled-release period. Approximately 27.9% of the drug payload was liberated within the first 15 min. This burst effect is likely attributed to the diffusion of BT molecules localized at or near the vesicular surface or intercalated within the superficial lipid interface. Subsequently, a gradual release was observed, with cumulative liberation reaching 53.6% at 1 h and 60.9% at 2 h, ultimately approaching a plateau of 78.8% by 12 h. In stark contrast, the BT solution exhibited near-instantaneous dissolution, with ~52% released by 15 min and complete exhaustion (~100%) achieved within 2 h. This significant divergence in release velocities underscores the robust sustained-release capacity of the AMS architecture. The protracted release from the optimized AMS formulation is mechanistically governed by the partitioning of BT within the concentric lipid bilayers and the aqueous core. These structures act as serial diffusional barriers, where the lipidic lamellae and the mucoadhesive SA moieties impose steric and electrostatic constraints that delay drug efflux into the bulk medium. In a clinical context, such controlled-release behavior is vital for maintaining the drug concentration within the therapeutic window for extended durations, thereby potentially mitigating the rapid washout associated with conventional ophthalmic drops and reducing daily dosing frequencies [82]. To elucidate the governing transport mechanism, the dissolution datasets were subjected to mathematical modeling, as presented in Table 5. The Korsmeyer–Peppas model yielded the highest correlation coefficient (R^2^ = 0.9537), with a calculated release exponent (n) of 0.5517, falling within the range for anomalous transport (0.45 < n < 0.89). The physical basis for anomalous (non-Fickian) transport from liposomal systems has been well described by Wu et al. [83], who showed that the Korsmeyer-Peppas model is uniquely fitted to explain time-dependent changes in drug release from liposomes under physiological conditions where Fickian diffusion through the bilayer is accompanied by membrane swelling/relaxation effects. The observed biphasic release profile of the optimized AMS formula fitted mechanistically with this finding. The lipid bilayer membrane of the developed AMS formula represents a diffusional barrier to the drug, while the SA-functionalized surface undergoes progressive hydration and swelling upon immersion in phosphate buffer (pH 7.4, 37 °C), contributing to the structural relaxation of the membrane. This swelling-driven erosion contributes to the non-Fickian release part of the release profile. The combination of these two mechanisms-diffusion and matrix relaxation- resulting in the anomalous transport described by the Korsmeyer-Peppas model.

### 3.5. Mucoadhesion Assessment

Mucin alone exhibited a markedly negative zeta potential (−17.8 ± 5.02 mV), which is characteristic of its anionic nature arising from sialic acid and sulfate residues on the mucin glycoprotein backbone. Following incubation of the cationic AMS with mucin, a pronounced shift in the zeta potential toward substantially lower negative charges was observed, with mean ZP value of −6.35 ± 3.16 mV. This charge attenuation can be mechanistically attributed to strong electrostatic attraction between the positively charged AMS and negatively charged mucin chains, leading to mucin adsorption and the formation of a mucin-coated aminosomal corona. The resulting shielding and overcompensation of surface charges confirm the establishment of stable AMS–mucin complexes. Such a zeta potential shift is a well-recognized mechanistic indicator of mucoadhesion and suggests enhanced interfacial interaction with mucosal layers, which is expected to prolong mucosal residence time and improve local drug retention [4,81].

### 3.6. Fourier Transform Infrared (FT-IR) Analysis

**Figure 3:**
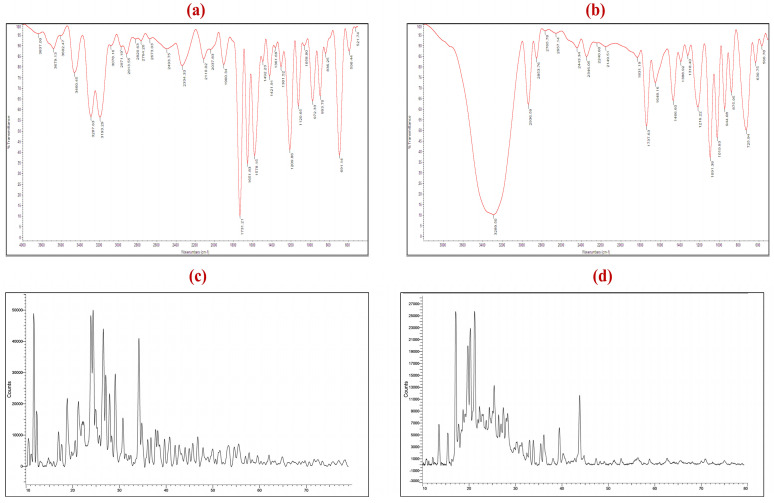
(**a**) FTIR spectrum of pure BT, (**b**) FTIR spectrum of BT-loaded AMS, (**c**) XRD spectrum of pure BT, and (**d**) XRD spectrum of BT-loaded AMS.

The FTIR spectrum of BT (Figure 3a) exhibited quintessential vibrational signatures indicative of its molecular architecture. Diagnostic absorption bands were identified at 3287.63 cm^−1^ and 3193.29 cm^−1^, corresponding to the N–H and O–H stretching vibrations, respectively. Furthermore, a sharp carbonyl (C=O) stretching vibration at 1731.21 cm^−1^ confirmed the presence of the tartrate moiety. The heterocyclic brimonidine ring was characterized by C=N and aromatic C=C stretching modes at 1651.83 cm^−1^ and 1578.15 cm^−1^, respectively. The presence of C–N and C–O stretching within the 1209.86–1120.65 cm^−1^ region corroborated the chemical integrity and purity of the drug candidate. In the vibrational profile of the BT-loaded AMS formulation (Figure 3b), the spectrum was predominantly characterized by the signals of the lipidic scaffold, indicating a transition in the drug’s microenvironment. The broadening and slight shift in the N–H/O–H stretching band (from 3287 to 3289.5 cm^−1^) indicate intermolecular hydrogen bonding, where the N–H groups of BT act as hydrogen bond donors interacting with phosphate oxygens (P=O and P–O–C) and ester carbonyl groups of phosphatidylcholine headgroups in lecithin, consistent with reported drug–liposome interaction patterns. The diagnostic peaks of BT at 1731.21 and 1651.83 cm^−1^ underwent significant attenuation and shifting to 1737.63 and 1648.16 cm^−1^, respectively. The apparent blueshift of the tartrate C=O band (from 1731.21 to 1737.63 cm^−1^) suggests a transition from the strongly hydrogen-bonded crystalline environment of pure BT to the relatively hydrophobic bilayer microenvironment of the vesicles, resulting in higher vibrational frequency of the carbonyl group. The attenuation of bands in the fingerprint region (1209.86–1120.65 cm^−1^), corresponding to C–N and C–O stretching vibrations of BT, likely reflects both the physical embedding of the drug within the lipid bilayer and the disruption of its crystalline lattice, consistent with the amorphization observed in XRD analysis. Importantly, the absence of new peaks indicates that these interactions are non-covalent, confirming the chemical compatibility of BT with the aminosomal matrix without evidence of chemical degradation or covalent adduct formation.

### 3.7. X-Ray Diffraction Analysis

The X-ray diffraction (XRD) pattern of pure BT (Figure 3c) exhibited numerous sharp, high-intensity Bragg reflections across the 2θ range of 10–80°, with prominent peaks in the 10–30° region characteristic of the monoclinic crystalline lattice of BT, consistent with reported crystallographic data [78]. In contrast, these characteristic reflections disappeared in the AMS formulation (Figure 3d) and were replaced by a broad diffuse halo, indicating the conversion of BT from a crystalline to an amorphous state. This amorphization can be attributed to two complementary mechanisms: spatial confinement of BT within the lipid bilayer aqueous core and interbilayer regions, which disrupts the long-range molecular order required for crystallization, and non-covalent drug–lipid interactions, particularly hydrogen bonding between BT N–H/O–H groups and lipid headgroups as supported by FTIR analysis. Additionally, during vesicle formation via ethanol injection, BT molecules become entrapped within nascent bilayer structures at concentrations below the nucleation threshold, preventing lattice formation. The resulting amorphous state is pharmaceutically advantageous, as amorphous forms generally exhibit higher apparent solubility and faster dissolution rates than crystalline counterparts [84], which likely contributes to the observed initial burst release (27.9% within 15 min) and supports successful molecular encapsulation of BT within the AMS matrix rather than surface deposition of crystalline drug.

### 3.8. In Vivo Evaluations of the Optimized Aminosomes Formulation

#### 3.8.1. Pharmacodynamic Evaluation of the IOP-Reducing Effect of the Optimized BT-Loaded Aminosome Formulation

**Figure 4:**
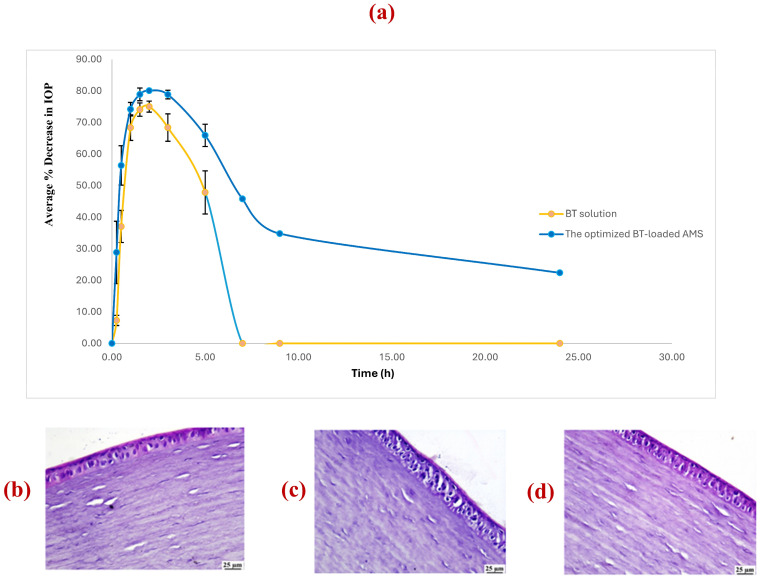
(**a**) Intraocular pressure reduction effect of BT from the optimized AMS formulation compared to BT solution; histopathological examination of the cornea after ocular administration of (**b**) normal saline (negative control); (**c**) BT solution; (**d**) the optimized AMS formulation.

The in vivo efficacy of the optimized AMS formulation relative to the standard BT aqueous solution was evaluated by monitoring the percentage reduction in intraocular pressure (IOP_red%_) over a 24 h period (Figure 4a). The optimized AMS formulation achieved a significantly higher maximum reduction in IOP (E_max_) (80.10 ± 1.53%) compared to the BT solution (75.04 ± 1.73%; *p* = 0.002). More notably, AMS demonstrated a profound augmentation in ocular bioavailability, evidenced by a 2.8-fold increase in the area under the curve (AUC_0–24h_) compared to the BT solution (970.59 ± 2.9%·h vs. 341.53 ± 5.3%·h, respectively; *p* < 0.0001). These data substantiate the superior capacity of the AMS carrier to enhance the transcorneal delivery of BT into the intraocular space. While both treatments reached peak efficacy at the same time point (T_max_ of 2 h), AMS exhibited a significantly accelerated onset of action. The optimized AMS formulation surpassed the 25% IOP reduction threshold within 15 min, a benchmark that required 30 min for the BT solution. This rapid onset suggests that the AMS architecture facilitates immediate interfacial interaction and rapid initial flux across the corneal epithelium.

A pronounced difference was observed in the duration of action, where AMS exhibited a 5.2-fold increase in mean residence time (MRT: 22.0 h vs. 4.20 h) and a 3.5-fold prolongation of biological half-life (T½: 15.73 h vs. 4.49 h) compared to the BT solution (*p* = 0.005 and *p* = 0.018, respectively). Specifically, the hypotensive effect of BT solution was transient, with IOP returning to baseline levels within 7 h while the IOP-lowering effect of AMS was robustly maintained throughout the 24 h study duration, concluding with a significant residual reduction of 22.39%.

The significant therapeutic gains observed with the AMS system are attributed to a synergistic tripartite mechanism involving biomimetic membrane fusion, permeation enhancement, and electrostatic mucoadhesion. Specifically, the structural homology between the AMS phospholipid bilayers and the corneal epithelial cell membranes facilitates vesicular fusion and lipid exchange, allowing the nanocarriers to serve as a biomimetic reservoir for the direct and sustained partition of BT into the corneal tissue. This process is augmented by the incorporation of phosphatidylcholine, which acts as a potent chemical penetration enhancer by transiently modulating the tight junctions of the corneal epithelium to facilitate increased transcorneal drug flux [58]. Furthermore, the inclusion of the cationic lipid SA enables high-affinity electrostatic tethering to the negatively charged mucin glycocalyx; this interfacial anchoring effectively resists nasolacrimal drainage, thereby maximizing precorneal residence time and maintaining the steep concentration gradient necessary to drive passive diffusion into the aqueous humor [25].

#### 3.8.2. Assessment of the Ocular Irritation by the Draize Test

To evaluate the safety profile of the optimized AMS architecture, a comparative in vivo ocular irritation study was conducted. Observation of the rabbit cohorts, Group A (optimized AMS formulation) and Group B (BT solution), revealed a total absence of inflammatory sequelae or visual abnormalities in the treated eyes. Systematic evaluation using the Draize scoring system yielded a cumulative score of zero for both the AMS formulation and the reference solution throughout the 24 h observation period. Specifically, no evidence of conjunctival hyperemia, chemosis, corneal opacity, or iritis was detected when compared to the contralateral untreated control eyes. These findings substantiate the biocompatibility and ophthalmic tolerability of the AMS system. The lack of irritation can be attributed to the use of biocompatible lipid components and the controlled concentration of the cationic agent (SA), ensuring that the mucoadhesive properties do not compromise the structural integrity of the ocular tissues. Consequently, the optimized AMS formulation is categorized as a non-irritant, making it a clinically viable candidate for chronic glaucoma management.

#### 3.8.3. Histopathological Assessment

To further substantiate the safety profile of the optimized nanocarrier, a comparative histopathological examination was performed on the ocular adnexa and intraocular tissues. Histomorphological analysis of the negative control group (normal saline) and the optimized AMS-treated group revealed an entirely preserved cytoarchitecture. As illustrated in Figure 4b,d, no pathological alterations, inflammatory cell infiltration, or structural derangements were observed across the cornea, retina, choroid, ciliary body, or conjunctiva. The corneal segments in the optimized AMS-treated group maintained a robust multi-layered epithelium, an organized stromal collagen matrix, and an intact endothelial lining, indicating that the cationic nature of the AMS does not induce epithelial desquamation or stromal edema.

In contrast, the eyes treated with the BT solution exhibited subtle microscopic deviations. Specifically, Figure 4c delineates mild vacuolation within the superficial squamous epithelial layer of the cornea. This focal vacuolar degeneration may be indicative of transient osmotic stress or direct drug-induced cytotoxic irritation when BT is presented in a free, non-encapsulated form. Notably, the posterior segments (retina, choroid, and ciliary body) in the BT solution group remained unremarkable, paralleling the control observations.

The absence of epithelial vacuolation in the optimized AMS-treated group, despite the presence of the same drug concentration, suggests that the lipid bilayer sequestration of BT within the AMS acts as a protective reservoir. By modulating the rate of drug exposure and preventing high-concentration hotspots of free drug on the corneal surface, the AMS architecture effectively mitigates the minor epithelial stress observed with the conventional solution. These findings provide definitive evidence that the optimized AMS are not only biocompatible but potentially safer for the ocular surface than standard aqueous formulations.

## 4. Conclusions

In this study, a mucoadhesive, stearylamine-enriched liposomal platform (Aminosomes) was successfully engineered for the ocular delivery of Brimonidine Tartrate (BT) using a refined ethanol injection technique. The systematic application of a full factorial design facilitated the precise interrogation of the design space, yielding an optimized formulation with superior critical quality attributes, including small particle size, narrow polydispersity, robust electropositive surface charge, and enhanced entrapment efficiency. Ultrastructural analysis via TEM corroborated the formation of discrete, monodisperse spherical nanovesicles, while FTIR and mucoadhesion assays substantiated the successful sequestration of the hydrophilic cargo and its strong biointerfacial affinity for ocular mucins. The in vitro kinetic profiles demonstrated a shift from immediate dissolution to a biphasic, sustained-release trajectory governed by anomalous non-Fickian transport. Most significantly, in vivo pharmacodynamic evaluations in a rabbit model revealed that the optimized AMS achieved a 2.8-fold increase in ocular bioavailability and a markedly prolonged hypotensive effect compared to conventional aqueous solutions. This pharmacokinetic superiority, characterized by an accelerated onset and a 24 h therapeutic duration, is attributed to the synergistic interplay of biomimetic membrane fusion, penetration enhancement, and electrostatic mucoadhesion. Furthermore, the absence of histopathological derangements or clinical irritation confirms the excellent biocompatibility and safety of the scaffold for chronic application. Collectively, these findings establish the BT-loaded AMS as a robust and clinically viable nanotherapeutic platform, offering a transformative approach to improving therapeutic adherence and efficacy in the long-term management of glaucoma and intraocular hypertension.

## Figures and Tables

**Table 1:** Overview of the factorial design: independent variables, recorded responses, and desirability targets.

**Factor (Independent Variable)**	**Levels**		
X_1_: Lecithin conc. (mg/mL)	15	30	45
X_2_: Lecithin to SA ratio	10:1	15:1	30:1
X_3_: BT amount (mg)	10		20
**Response (Dependent Variable)**	**Desirability Constraints**		
Y_1_: PS (nm)	Minimize		
Y_2_: PDI	Minimize		
Y_3_: ZP (mV)	Maximize		
Y_4_: EE%	Maximize		

Abbreviations: BT, Brimonidine Tartrate; EE, entrapment efficiency; PS, particle size; PDI, polydispersity index; SA, stearylamine; ZP, zeta potential.

**Table 2:** The compositions and in vitro characterization results of the BT-loaded Aminosomes formulations prepared based on the 3^2^ × 2^1^ full factorial design.

Formulae	Lecithin Concentration (mg/mL)	Lecithin to SA Ratio	BT Amount (mg)	PS (nm) *	PDI *	ZP (mV) *	EE (%) *
F1	15	10:1	10	144.60 ± 1.69	0.16 ± 0.00	43.50 ± 2.82	28.70 ± 0.91
F2	15	15:1	10	184.10 ±2.54	0.21 ± 0.00	26.05 ± 2.19	40.22 ± 5.03
F3	15	30:1	10	181.65 ± 4.31	0.29 ± 0.04	11.30 ± 0.14	35.84 ± 0.36
F4	30	10:1	10	405.85 ± 0.91	0.27 ± 0.20	23.27 ± 0.31	35.83 ± 6.38
F5	30	15:1	10	208.60 ± 0.28	0.261 ± 0.01	20.60 ± 1.27	35.60 ± 1.72
F6	30	30:1	10	193.40 ± 6.93	0.31 ± 0.06	21.65 ± 0.49	48.65 ± 0.19
F7	45	10:1	10	314.55 ± 2.75	0.48 ± 0.01	57.15 ± 1.90	49.94 ± 2.10
F8	45	15:1	10	218.60 ± 0.42	0.37 ± 0.04	45.65 ± 1.48	52.21 ± 7.32
F9	45	30:1	10	278.45 ± 4.03	0.29 ± 0.06	38.85 ± 0.21	53.42 ± 0.18
F10	15	10:1	20	153.40 ± 0.84	0.22 ± 0.01	63.95 ± 4.17	60.30 ± 5.34
F11	15	15:1	20	162.40 ± 5.37	0.19 ± 0.01	50.60 ± 0.14	48.61 ± 0.18
F12	15	30:1	20	168.90 ± 2.12	0.22 ± 0.00	37.90 ± 0.98	42.01 ± 0.73
F13	30	10:1	20	239.25 ± 2.75	0.30 ± 0.02	42.55 ± 0.35	42.21 ± 7.65
F14	30	15:1	20	207.30 ± 2.54	0.33 ± 0.00	42.30 ± 0.42	45.92 ± 2.87
F15	30	30:1	20	189.20 ± 4.52	0.23 ± 0.01	13.50 ± 0.28	53.00 ± 0.18
F16	45	10:1	20	460.15 ± 2.37	0.56 ± 0.02	53.45 ± 5.40	58.57 ± 0.17
F17	45	15:1	20	446.80 ± 5.23	0.46 ± 0.00	48.35 ± 0.07	55.93 ± 0.80
F18	45	30:1	20	482.95 ± 9.86	0.23 ± 0.15	38.95 ± 0.07	57.58 ± 4.09

* Data expressed as means ± SD (*n* = 3). BT, Brimonidine Tartrate; EE, entrapment efficiency; PS, particle size; PDI, polydispersity index; SA, stearylamine; ZP, zeta potential.

**Table 3:** The fit statistics of the selected models for the studied responses in the 3^2^ × 2^1^ full factorial design.

Response	Model	*p*-Value	R^2^	Adjusted R^2^	Predicted R^2^	Adequate Precision	Significant Factors
Y_1_: PS (nm)	2FI linear	<0.0001	0.7423	0.6890	0.6341	11.0941	A and C
Y_2_: PDI	2FI linear	<0.0001	0.7942	0.7516	0.6752	15.0014	A and B
Y_3_: ZP (mV)	Quadratic	<0.0001	0.9066	0.8789	0.8378	19.5772	A, B, and C
Y_4_: EE%	Quadratic	<0.0001	0.7181	0.6345	0.5004	8.6230	A and C

EE, Entrapment efficiency; PS, particle size; PDI, polydispersity index; 2FI, two-factor interaction; ZP, zeta potential.

**Table 4:** The predicted and the actual responses of the optimized Aminosomes formula.

Responses	PS (nm)	PDI	ZP (mV)	EE (%)
Predicted	125.94	0.19	64.22	52.15
Observed	153.40 *	0.22 *	63.95 *	60.30 *
% Deviation	17.90	13.64	0.42	13.51

* Data represented as means (*n* = 3). EE, Entrapment efficiency; PS, particle size; PDI, polydispersity index; ZP, zeta potential.

**Table 5:** Release kinetic modeling parameters for BT from the optimized AMS formulation.

Kinetic Model	Zero-Order	First-Order	Higuchi	Hixson–Crowell	Korsmeyer–Peppas
Determination Coefficient (R^2^)	0.6562	0.4704	0.9432	0.723	0.9537

## Data Availability

The datasets generated and analyzed during the current study are included in this published article.
